# The Segment Anything foundation model achieves favorable brain tumor auto-segmentation accuracy in MRI to support radiotherapy treatment planning

**DOI:** 10.1007/s00066-024-02313-8

**Published:** 2024-11-06

**Authors:** Florian Putz, Sogand Beirami, Manuel Alexander Schmidt, Matthias Stefan May, Johanna Grigo, Thomas Weissmann, Philipp Schubert, Daniel Höfler, Ahmed Gomaa, Ben Tkhayat Hassen, Sebastian Lettmaier, Benjamin Frey, Udo S. Gaipl, Luitpold V. Distel, Sabine Semrau, Christoph Bert, Rainer Fietkau, Yixing Huang

**Affiliations:** 1 Department of Radiation Oncology, Universitätsklinikum Erlangen, Friedrich-Alexander-Universität Erlangen-Nürnberg, Universitaetsstraße 27, 91054 Erlangen, Germany; 2 Comprehensive Cancer Center Erlangen-EMN (CCC ER-EMN), Erlangen, Germany; 3 The Bavarian Cancer Research Center (BZKF), Erlangen, Germany; 4 Institute of Neuroradiology, University Hospital Erlangen, Friedrich-Alexander-Universität Erlangen-Nürnberg, Erlangen, Germany; 5 Institute of Radiology, University Hospital Erlangen, Friedrich-Alexander-Universität Erlangen-Nürnberg, Erlangen, Germany

**Keywords:** Deep learning, Interactive segmentation, Glioma, Tumor auto-contouring, Artificial intelligence

## Abstract

**Background:**

Promptable foundation auto-segmentation models like Segment Anything (SA, Meta AI, New York, USA) represent a novel class of universal deep learning auto-segmentation models that could be employed for interactive tumor auto-contouring in RT treatment planning.

**Methods:**

Segment Anything was evaluated in an interactive point-to-mask auto-segmentation task for glioma brain tumor auto-contouring in 16,744 transverse slices from 369 MRI datasets (BraTS 2020 dataset). Up to nine interactive point prompts were automatically placed per slice. Tumor boundaries were auto-segmented on contrast-enhanced T1w sequences. Out of the three auto-contours predicted by SA, accuracy was evaluated for the contour with the highest calculated IoU (Intersection over Union, “oracle mask,” simulating interactive model use with selection of the best tumor contour) and for the tumor contour with the highest model confidence (“suggested mask”).

**Results:**

Mean best IoU (mbIoU) using the best predicted tumor contour (oracle mask) in full MRI slices was 0.762 (IQR 0.713–0.917). The best 2D mask was achieved after a mean of 6.6 interactive point prompts (IQR 5–9). Segmentation accuracy was significantly better for high- compared to low-grade glioma cases (mbIoU 0.789 vs. 0.668). Accuracy was worse using the suggested mask (0.572). Stacking best tumor segmentations from transverse MRI slices, mean 3D Dice score for tumor auto-contouring was 0.872, which was improved to 0.919 by combining axial, sagittal, and coronal contours.

**Conclusion:**

The Segment Anything foundation segmentation model can achieve high accuracy for glioma brain tumor segmentation in MRI datasets. The results suggest that foundation segmentation models could facilitate RT treatment planning when properly integrated in a clinical application.

## Introduction

Tumor segmentation in MRI constitutes the essential initial phase in radiotherapy treatment planning for patients with brain tumors [1, 2]. Owing to the imaging heterogeneity of tumors, this indispensable image processing step still predominantly relies on time-consuming manual contouring by physicians [2]. The substantial time demand of manual contouring restricts the accessibility of precise target volume definition, particularly in countries with limited resources [3]. Deep neural networks, such as U‑net variants, have demonstrated exceptional accuracy in tumor auto-segmentation in research studies [4, 5]. However, implementing these models for specific tumor types and unique imaging data typically necessitates large, dedicated, expert-segmented training datasets that are representative of the data to which the model will be applied later [6]. Since out-of-distribution errors and poor generalizability can pose risks to treatment planning [7], automatic deep-learning-based tumor auto-segmentation has not yet experienced widespread clinical adoption. Furthermore, conventional deep learning auto-segmentation models generally lack the capacity for expert–model interaction, which is necessary to efficiently perform corrections and adaptations to the model output under the guidance of clinical experts.

Segment Anything (Meta AI, New York, USA) belongs to the novel class of promptable foundation models for auto-segmentation and has been trained on 11 million conventional 2D photographs and over 1 billion object segmentation masks [8]. The model enables promptable auto-segmentation in which the model output segmentation masks can be controlled using a variety of input prompts, such as foreground and background points, input boxes and masks, and text prompts. The Segment Anything model comprises a prompt encoder and an image encoder, both responsible for encoding the prompt and image input, and a mask decoder that connects to both encoders to predict valid segmentation masks. Interestingly, Segment Anything has been designed to predict three masks per prompt input to be able to deal with the common ambiguity of input prompts [8].

Segment Anything has shown high zero-shot auto-segmentation performance for a large variety of 2D images from many different categories, including plant photography, traffic cameras, underwater scenes, and microscopy; however, its performance on medical imaging data for radiotherapy treatment planning remains largely unexplored [8].

In the present manuscript, we investigate the Segment Anything foundation model in brain tumor auto-segmentation on MRI datasets. Our work has a dual objective: first, we evaluate the segmentation accuracy of the Segment Anything model for treatment planning in glioma patients for the purpose of an expert-interactive workflow. Second, by assessing the zero-shot performance on brain tumor MRI datasets, we aim to further examine the generalizability of the Segment Anything foundation model for datasets that significantly deviate from the original training set.

### Contributions

In this manuscript, we make three core contributions. (1) We establish that Segment Anything can attain high segmentation accuracy for brain tumor MRI datasets in an interactive point-to-mask application. (2) We consequently demonstrate that Segment Anything effectively generalizes to brain tumor MRI datasets, achieving a segmentation accuracy comparable to that observed in the 2D photographs using which it was previously evaluated. (3) We identify challenges encountered when applying Segment Anything to tumor segmentation in MRI datasets and identify strategies to address these challenges, which are also applicable in the context of clinical implementation.

## Methods

### Brain tumor MRI dataset

In this study, we utilize the open BraTS 2020 glioma segmentation MRI dataset for evaluation purposes [9]. BraTS is a renowned glioma segmentation challenge in which research groups compete using their segmentation models. The BraTS 2020 segmentation challenge encompasses a training dataset consisting of 369 volumetric MRI studies and a validation set comprising 125 studies. Each study includes a volumetric pre-contrast and contrast-enhanced T1-weighted MRI sequence (T1ce), a T2-weighted sequence, and a T2-FLAIR-weighted sequence [10]. As only the training dataset provides ground truth segmentation masks, rendering it suitable for automatic evaluation of an interactive point-to-mask auto-contouring task analogous to the methodology employed in the original Segment Anything paper, we exclusively utilize the training dataset for our present evaluation [8].

The BraTS 2020 training set contains 259 datasets of patients with high-grade glioma and 110 datasets of low-grade gliomas. All datasets in the BraTS 2020 training set are already provided as spatially normalized and skull stripped and resampled to a common voxel grid of 240 × 240 × 155 with 1‑mm isotropic resolution. The ground truth segmentation masks comprise three classes: necrotic tumor core, contrast-enhancing tumor, and peritumoral edema. For evaluation purposes, these classes are combined in the BraTS challenge into tumor core (necrotic core and contrast-enhancing tumor), whole tumor (tumor core + peritumoral edema), and enhancing tumor [5, 10]. To assess Segment Anything for its potential in supporting interactive clinical treatment planning and because the model is limited to a single image input, we evaluate segmentation accuracy using a single MRI sequence as input. As tumor core frequently represents the only relevant tumor compartment for clinical treatment planning [1, 11] and as edema in the BraTS 2020 dataset was defined using multiple sequences, we exclusively examine tumor core segmentation on contrast-enhanced T1-weighted sequences.

### Evaluation of auto-segmentation performance

We assess the Segment Anything segmentation performance on 2D MRI slices using a dedicated automated evaluation pipeline implemented in Python for 3D Slicer (v. 5.22) [12]. Inference was executed locally with the Segment Anything version 1.0 program code and ViT‑H model weights on a workstation equipped with an NVIDIA RTX A6000 GPU, featuring 48 GB GPU memory (Python 3.9.10, PyTorch 1.10.1 with CUDA 11.1). MRI voxel intensities were normalized to a range between 0 and 255 by dividing them by the maximum intensity of each 3D dataset and subsequently multiplying by 255. Since Segment Anything anticipates a three-channel image input, the rescaled intensities were replicated across all three channels. The automated point-to-mask auto-segmentation performance evaluation involved the following steps:

1. Each transversal 2D MRI slice containing tumor core voxels was evaluated.
2. The initial point prompt was positioned at the tumor center on the respective slice (highest distance transform), and subsequent points were placed at the center of the set difference between the ground truth segmentation and the mask prediction, with a maximum of nine interactive input points placed.
3. If the ground truth segmentation area exceeded the mask prediction, a foreground point was placed at the center of the set difference (ground truth–predicted mask); otherwise, a background point was placed at the center of the set difference (predicted mask–ground truth).
4. For each iteration, the intersection over union (IoU) between the ground truth mask and the predicted mask was calculated.

The best achieved IoU per slice was evaluated as the primary performance metric, reflecting an expert-interactive workflow in which the expert would retain the optimal mask prediction. To address input prompt ambiguity, Segment Anything produces three masks per prediction, along with an IoU estimate for each mask. We assessed two types of mask predictions: (1) the mask with the highest predicted IoU (suggested mask) and (2) the mask with the highest calculated IoU relative to the ground truth (oracle mask). In this evaluation, we primarily utilized the oracle mask, as it best represents an expert-interactive workflow where the expert selects the most accurate mask prediction.

In addition to evaluating Segment Anything on whole MRI slices, we also assessed model performance on cropped images using a cuboid region-of-interest (ROI) fitted to the 3D tumor extent with a 2-cm margin. For each experiment and both oracle and suggested masks, we evaluated a total of 16,744 2D transversal MRI slices. To facilitate comparability with 3D segmentation methods, 2D slice-based predictions were stacked, and the volumetric Dice score was calculated relative to the ground truth. Lastly, 3D segmentations from stacked slice-based predictions from transverse, coronal, and sagittal slices were combined using majority voting to provide an indication of the expected performance in an expert-interactive workflow where multiple slice orientations could be employed.

Statistical analyses were performed using SPSS (version 21.0.0.2; IBM Corp., Armonk, NY, USA) and R (version 3.5.2; R Project for Statistical Computing, R Foundation, Vienna, Austria), with graphs created using SPSS and GraphPad Prism (version 9.5.0; GraphPad Software, La Jolla, CA, USA). The optimum threshold for the number of tumor voxels per MRI slice with regard to segmentation accuracy was calculated using maximally selected rank statistics (R package maxstat, Wilcoxon statistics, *p*-value adjusted for multiple testing). Differences between paired experiments were compared using a Wilcoxon signed-rank test, while unpaired experiments employed a Wilcoxon rank-sum test. *P*-values <0.05 were deemed statistically significant.

## Results

### Evaluation on whole MRI slices

**Fig. 1:**
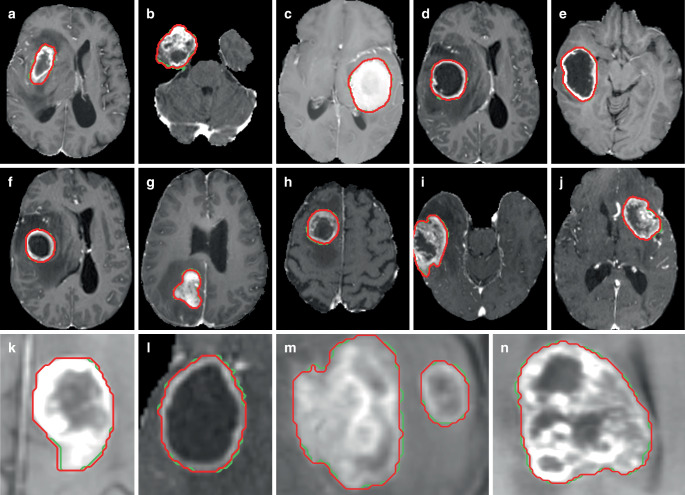
Exemplary Segment Anything glioma auto-segmentations on contrast-enhanced T1 sequences from the BraTS 2020 dataset. *Green* ground truth segmentation,* red* best Segment Anything oracle mask tumor auto-segmentation obtained with up to nine automated interactive point prompts. **a**–**j** Full brain views of transversal MRI slices, **k**–**n **enlarged view

**Fig. 2:**
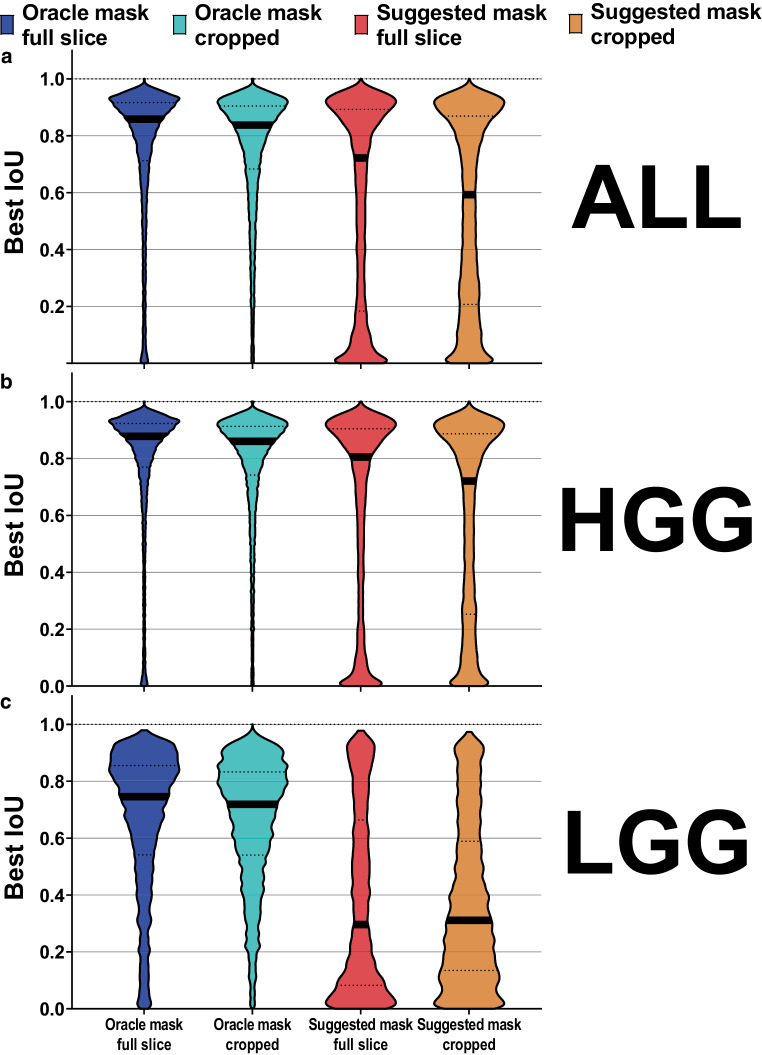
Mean best intersection over union (*IoU*) obtained with Segment Anything point-to-mask auto-segmentation in the BraTS 2020 training dataset (*n* = 369 contrast-enhanced T1 datasets, 16,744 MRI slices). *Blue* predicted oracle mask on full MRI slices, *cyan* predicted oracle mask on MRI slices cropped to the 3D tumor extent +2-cm margin, *red* most confident model prediction (suggested mask) on full MRI slices, *orange* suggested mask on cropped slices. **a** Results for all datasets, **b** results for high-grade glioma (*HGG*) datasets, **c** results for low-grade glioma (*LGG*) datasets. Note: decrease in accuracy with LGG compared to HGG cases

A total of 369 MRI datasets and 16,744 MRI slices were evaluated (Fig. 1). Out of all 16,744 MRI slices evaluated, 13,035 slices were images of high-grade gliomas (HGGs) and 3709 were images of low-grade gliomas (LGGs). Mean best intersection over union (IoU) per slice for the oracle mask tumor contour was 0.762 (interquartile range [IQR] 0.713–0.917). Interestingly, mean best IoU was significantly better for HGG compared to LGG cases: 0.789 (IQR 0.770–0.923) vs. 0.668 (IQR 0.541–0.855; *p* < 0.001; Fig. 2). The mean number of interactive point prompts required to obtain the best tumor contour mask prediction was 6.6 (IQR 5–9).

### Change in accuracy with the number of input point prompts

**Fig. 3:**
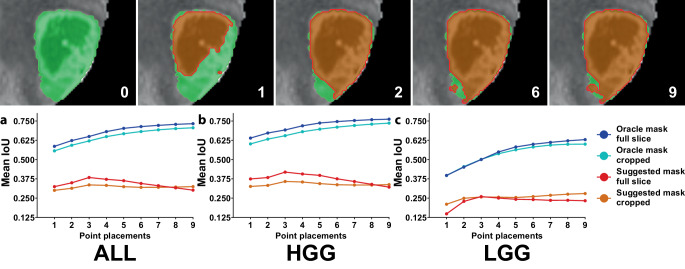
Segment Anything segmentation accuracy in relation to the number of interactive input point prompts. **a** An example case illustrating the increased similarity between the ground truth segmentation and the Segment Anything mask prediction as the number of interactive point placements increases (*green *ground truth segmentation, *red *auto-segmentation [oracle mask, full MRI slices]). **b** Mean intersection over union (*IoU*) for 1–9 point prompts for the entire dataset (*ALL*; **a**), high-grade (*HGG*; **b**), and low-grade glioma cases (*LGG*; **c**). Note: an IoU improvement for an increasing number of input point prompts is observed for the predicted oracle tumor mask but not for the model-suggested mask

Single point-to-mask segmentation IoU was 0.586 (IQR 0.314–0.864) for the oracle mask tumor contour. For HGGs, single point accuracy was 0.640 (IQR 0.465–0.880) but only 0.397 (IQR 0.110–0.669) for LGGs (*p* < 0.001). Mean IoU using the oracle mask tumor contour consistently improved with the number of interactive point prompts for HGG as well as LGG cases (Fig. 3).

### Common segmentation errors

**Fig. 4:**
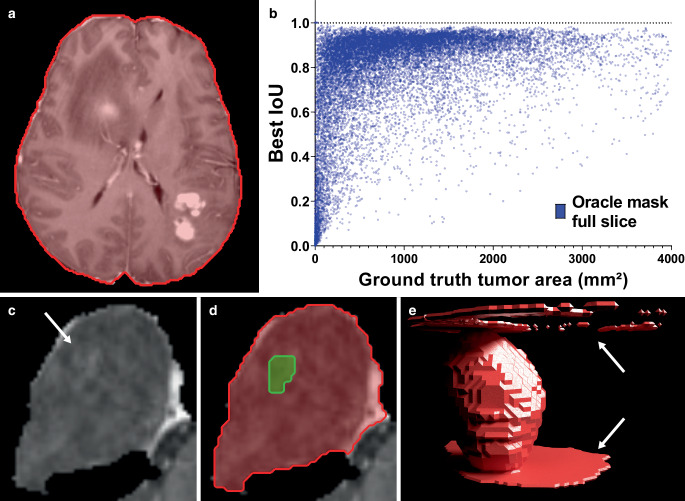
Common auto-segmentation errors observed with Segment Anything on T1 contrast-enhanced glioma MR images. **a** Whole patient/brain circumference is segmented by Segment Anything instead of the tumor, because of prompt ambiguity. **b** Scatter plot of mean best IoU as a function of ground truth tumor area. Note: low-accuracy segmentations are primarily observed for MR images with few tumor voxels. **c–e** Example case illustrating problems with segmentation on peripheral slices with few tumor voxels after single point placement

Figure 4 shows examples of common segmentation errors. Because of prompt ambiguity, Segment Anything sometimes segmented the whole patient/brain circumference instead of the tumor. This was especially observed, for single point prompts, for the most confident (suggested mask) instead of the oracle mask tumor contour and at peripheral slices with few tumor voxels. Segmentation errors were mostly observed in peripheral slices that only contained few tumor voxels (Fig. 4b and c–e). Figure 4b illustrates that low segmentation accuracy clustered to slices with few tumor voxels and that there was a correlation between the mean best IoU and the ground truth tumor area (Spearman ρ = 0.495, *p* < 0.001). A minimum tumor area of 300 mm^2^ per slice was the optimal threshold separating slices with high and low segmentation accuracy by maximally selected rank statistics. Mean best IoU for oracle mask was 0.841 (IQR 0.804–0.926) for a minimum tumor area of 300 mm^2^, whereas it was only 0.537 (IQR 0.244–0.821) for a tumor area <300 mm^2^ (adjusted *p* < 0.001). Single point-to-mask mean IoU was 0.670 (IQR 0.499–0.883) vs. 0.346 (IQR 0.018–0.699) for tumor areas ≥ and <300 mm^2^, respectively. The highest single point-to-mask auto-contouring accuracy for the oracle mask tumor contour was observed for the subgroup of high-grade gliomas with a tumor area ≥ 300 mm^2^ (0.735).

### Evaluation on MRI slices cropped to the tumor region

Since segmentation errors involving the entire patient circumference were frequently observed, we also assessed Segment Anything on image patches cropped to the 3D tumor circumference with an additional 2‑cm margin. This could easily be integrated into an expert interactive workflow and would correspond to the expert zooming in on the tumor region. However, contrary to expectations, cropping did not enhance segmentation accuracy but rather diminished it (Figs. 2 and 3). The mean best IoU was 0.762 (IQR 0.713–0.917) without cropping and 0.759 (IQR 0.683–0.905) with cropping (*p* < 0.001). Furthermore, the oracle tumor mask consistently displayed poorer performance when applying cropping as opposed to not cropping across all numbers of input point prompts (Fig. 3b). A similar decline in performance was observed for whole-slice image input with an additional, equally sized box prompt, and even for a box prompt precisely fitted to the 3D tumor extent. Box prompts tailored to individual 2D slices were not examined, as combining box and point prompts on each individual slice was considered too time consuming for a clinical workflow.

### Results for model-suggested mask prediction

Out of the three tumor contours predicted by Segment Anything, the highest-confidence tumor auto-contour (suggested mask) consistently exhibited substantially lower performance compared to the oracle mask (Figs. 2 and 3). The mean best IoU for the oracle tumor mask without cropping was 0.762 (IQR 0.713–0.917), while for the model-suggested mask, it was only 0.572 (0.184–0.892; *p* < 0.001). Intriguingly, no consistent improvement for the model-suggested mask was observed with increasing number of input point prompts (Fig. 3).

### 3D segmentation accuracy with and without combining multiple slice orientations

**Fig. 5:**
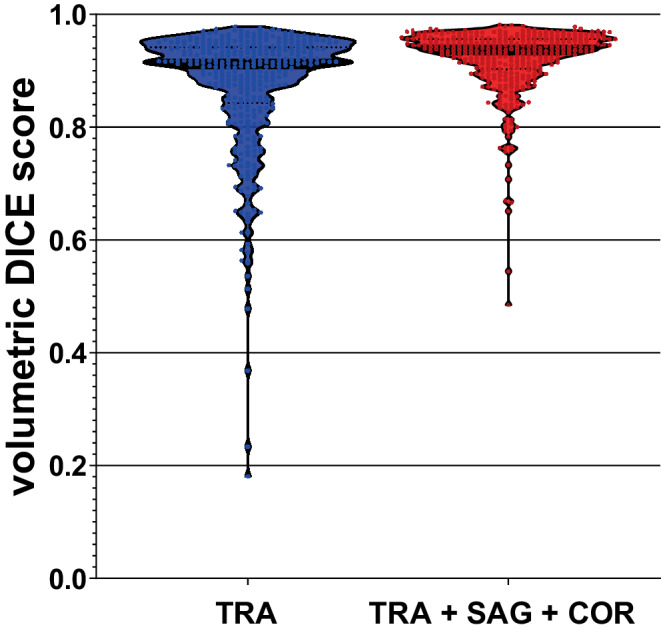
Volumetric Dice score achieved by stacking Segment Anything 2D tumor contours (best oracle mask prediction per slice, full MRI slices). *Blue* Segment Anything segmentation using only transverse slices (*TRA*); *red* Segment Anything segmentation employing transversal, sagittal, and coronal slices (*TRA + SAG + COR*) combined via simple majority voting, which corresponds to a potential interactive clinical workflow where an expert can utilize all three slice orientations. Note: significant improvement in 3D segmentation accuracy by combining all three slice orientations

To evaluate the accuracy of Segment Anything point-prompt-based interactive auto-contouring in comparison to other methods published for glioma auto-segmentation, we calculated the volumetric Dice similarity score by stacking the Segment Anything 2D tumor contours. For all 369 BraTS training datasets, the mean volumetric tumor core Dice score achieved by stacking the best oracle mask 2D segmentations from transversal slices was 0.872 (IQR 0.843–0.941). As segmentation errors were predominantly observed for peripheral slices (see above), the mean volumetric Dice score could be further improved to 0.919 (IQR 0.903–0.956; *p*-value for difference <0.001) by combining Segment Anything 2D masks from transverse, coronal, and sagittal slice orientations (Fig. 5). This approach would correspond to a clinical expert utilizing Segment Anything across multiple slice orientations in an interactive workflow.

## Discussion

The foundation auto-segmentation model Segment Anything achieved high accuracy for interactive glioma auto-contouring in this evaluation. The volumetric Dice score for tumor segmentation achieved by stacking 2D tumor contour predictions from transverse slices was 0.872 and even reached 0.919 by combining 2D masks from transverse, sagittal, and coronal slice orientations. In contrast, the winners of the BraTS 2020 glioma auto-segmentation challenge—Isensee et al. using an optimized nnU-net implementation—reported a tumor core Dice score of 0.851 in the BraTS 2020 validation set [5, 9]. The two evaluations may not be directly compared, as the tasks was completely different (fully automatic 3D segmentation after supervised learning vs. zero-shot point-to-2D mask) and the evaluation dataset differed (BraTS 2020 training dataset vs. validation dataset). However, the juxtaposition illustrates that the Segment Anything foundation model could indeed achieve a high segmentation accuracy in an expert-interactive workflow.

The generalizability of the Segment Anything foundation segmentation model to brain tumor MRI datasets can be considered remarkable, as the model was solely trained for object segmentation in 2D photographs. Image slices from 3D MRI datasets are acquired based on completely different physical principles to conventional 2D photographs [13]. Moreover, glioma brain tumors are not discrete objects in three-dimensional space but are highly infiltrative tumors that are to some extent continuous with their surroundings [14–16]. The segmentation accuracy observed for Segment Anything in brain tumor MR images was slightly worse than for 2D photographs as reported by the Segment Anything authors [8]. In their seminal paper, Segment Anything was evaluated across 23 datasets with 2D photographs ranging from plant images, aerial photographs, traffic camera footage, and cooking photos to images of recycling waste and underwater scenes [8]. The Segment Anything single point-to-mask segmentation accuracy was slightly below the IoU of 0.750 for 2D photographs (value obtained from plot), whereas it was 0.586 in the present evaluation on glioma auto-contouring. However, in the present evaluation, the accuracy was higher for high-grade than for low-grade gliomas, was dragged down by peripheral slices with low tumor area, and consequently reached 0.735 on high-grade glioma MRI slices with at least 300 tumor voxels. Moreover, the segmentation accuracy increased consistently with an increasing number of interactive input point prompts for the oracle mask tumor contour predictions.

To be able to handle prompt ambiguity, Segment Anything makes three mask predictions per input image [8]. In this manuscript, we evaluated the objectively best tumor contour prediction (oracle mask) that had the best calculated IoU as the main evaluation metric. In addition, we also evaluated the most confident tumor contour prediction (suggested mask) that had the highest model-predicted IoU. In stark contrast to the oracle mask tumor contour accuracy, segmentation performance for the model-suggested mask was very low and did not consistently improve with the number of input point prompts (Fig. 3). This finding could indicate that the Segment Anything IoU predictions do not perform well in the context of medical datasets and segmentation tasks.

The reliance on oracle mask predictions could be seen as a disadvantage for implementation in a clinical application, as for conventional 2D photographs this would correspond to the interacting user having to evaluate all three mask predictions for every image. However, because of the redundancy of high-resolution medical 3D datasets that change very little from one slice to another, the finalized segmentation mask of the previous slice is a valid approximation for the ground truth mask of each slice. Thus, with sequential slice-wise segmentation, the mask prediction most similar to the previous slice segmentation can be automatically selected to achieve a similar performance as obtained with the oracle mask prediction in this evaluation. Therefore, the evaluation performed in the current work can readily be integrated into a clinical workflow. To provide a practical example for a clinical implementation of Segment Anything and to facilitate further clinical assessments, we have developed and share a corresponding segmentation extension for the open-source software 3D Slicer* [12].

Because of the heterogeneity of tumors and imaging data, deep learning tumor auto-segmentation has not yet seen widespread clinical adoption for treatment planning. Dedicated deep learning auto-segmentation models trained for specific tumor types in specific medical imaging datasets, however, have achieved high accuracy in the context of scientific studies [4, 5, 17–19]. For brain tumors, these models have shown very promising performance for glioma segmentation, but also for brain metastases and vestibular schwannomas [5, 6, 17, 18, 20]. For brain metastases, Lu et al. were able to demonstrate in a multi-reader study that assistance from a deep learning ensemble auto-segmentation model (3D U‑Net + DeepMedic) could improve brain metastasis detection sensitivity from 82.6% to 91.3% and the inter-reader Dice score from 0.86 to 0.90 compared to manual expert evaluation alone [21]. 3D and 2D U‑net variants have shown particularly high auto-segmentation performance and have undergone widespread evaluation and adaption for a vast number of biomedical segmentation tasks [4, 19, 22, 23]. Because of the great success of dedicated auto-segmentation models trained in a supervised fashion for specific medical segmentation tasks, more widespread introduction of these models into clinical radiotherapy treatment planning is only a question of time.

However, large promptable foundation models for interactive auto-segmentation like Segment Anything that show high zero-shot accuracy and can generalize to a vast number of segmentation problems should also have an important role to play in RT treatment planning. Dedicated deep learning auto-segmentation models like U‑net variants trained in a conventional supervised fashion can usually only be applied to a very narrow range of tumor types and image datasets that correspond to a preexisting training dataset [4]. While such dedicated models can be created for frequently encountered tumor and image dataset types like, e.g., brain metastases in contrast-enhanced 3D-T1w gradient echo sequences, a substantial proportion of segmentation problems will likely remain for which the generation, evaluation, and regulatory approval of dedicated segmentation models in the present form will not be economically feasible. Moreover, dedicated deep learning auto-segmentation models showing high segmentation accuracy at a cohort level can still fail for very specific patient cases and unseen image datasets because of limited generalizability, making expert review and correction of auto-segmentations mandatory [7]. Finally, slice-based 2D promptable auto-segmentation models like Segment Anything could also greatly accelerate the creation of annotated training datasets for supervised training of dedicated deep learning auto-segmentation models.

Most deep learning tumor auto-segmentations necessitate some degree of expert correction to obtain the final segmentation for treatment planning. Since dedicated deep learning auto-segmentation models do not allow for user interaction, correcting auto-segmentations typically involves fully manual slice-wise adjustments to the segmented tumor boundary, which is highly inefficient [24]. Drawing an analogy, this sequence of tumor auto-segmentation and manual correction is akin to traveling from one city to another by plane, only to walk the remaining distance from the airport to the destination by foot. Therefore, the novel class of promptable foundation image segmentation models is ideally suited for expert-interactive segmentation tasks and interactive correction of auto-segmentations. Segment Anything allows for multiple types of input prompts, which would allow clinical experts to make rapid adaptions and corrections to segmentations. The present evaluation clearly demonstrates this adaptation of mask predictions towards the ground truth segmentation (e.g., Fig. 3). In addition to foreground and background point prompts, which were mainly evaluated in the present work, Segment Anything also allows for box, mask, and text input prompts [8]. Additional box prompts were not helpful in the present evaluation and were therefore not further pursued. However, box prompts should be enabled in clinical implementations for an expert-interactive workflow to give the user further freedom for interaction with the auto-segmentation model, which should be helpful for certain segmentation problems. Finally, mask and text prompts were not evaluated in the present manuscript. While introducing text prompts for medical segmentation problems is highly interesting, we did not include it in this initial evaluation, as text prompts would be very difficult to implement in an automated evaluation and as we expected the CLIP-based text prompt implementation to perform poorly with medical nomenclature on MRI datasets. Finally, mask-based prompts were not evaluated in the present work, as mask input in Segment Anything is currently limited to low-resolution logit masks, and drawing input masks for individual slices would not accelerate clinical treatment planning in a meaningful way [8]. However, mask input in the context of medical 3D dataset segmentation is interesting and could, e.g., allow propagation of 2D segmentations across multiple slices.

The fact that Segment Anything only allows for 2D segmentations can be seen as a disadvantage for segmenting 3D medical datasets. While a similar promptable foundation model for 3D segmentation in volumetric medical datasets would certainly be greatly beneficial for treatment planning, 2D auto-segmentation has the advantage that every image slice is segmented under expert guidance in an interactive way and fits very well into current applications for radiotherapy treatment planning. Aside from facilitating and accelerating treatment planning, the high 2D segmentation accuracy of segmentation foundation models could also support diagnostic applications like automatic perpendicular diameter measurements that currently form the foundation of response assessment in gliomas [25].

Although the auto-contouring performance and generalizability of Segment Anything for the task of glioma brain tumor auto-segmentation in MRI datasets is already good, additional performance improvements may be achieved by fine-tuning segmentation foundation models using medical datasets. The present evaluation supports this assumption, as common segmentation errors included whole brain area segmentation and inaccurate IoU predictions for the three output masks.

Recent breakthroughs in deep learning models like GPT‑4 (OpenAI, San Francisco, USA) and Segment Anything, which demonstrate unparalleled generalization abilities, have resulted from massive training datasets [8, 26]. Segment Anything was trained on 11 million images and over one billion masks [8]. Although details of GPT-4’s training data remain undisclosed, they are likely to encompass a significant portion of the internet’s available text data [26].

Regrettably, extensive medical datasets of a similar scale are not yet available. It is therefore advantageous that large foundation models like Segment Anything, trained on vast non-medical datasets, can be meaningfully transferred to the medical domain, potentially including further fine-tuning on smaller medical datasets.

## Conclusion

The Segment Anything promptable foundation segmentation model demonstrated high accuracy for interactive glioma auto-contouring in T1ce MRI datasets. These findings indicate that auto-segmentation foundation models could accelerate and facilitate RT treatment planning when properly integrated into a clinical application. Furthermore, the results highlight the ability of large promptable foundation segmentation models to generalize effectively to medical imaging data.
